# Field validation of effects of species and flock size on echoes in avian radar surveys

**DOI:** 10.1038/s41598-024-73198-x

**Published:** 2024-09-29

**Authors:** Taito Kamata, Takahiro Sato, Koki Tateishi, Kayo Koumura, Yoichi Kawaguchi, Tsuneo Sekijima

**Affiliations:** 1 Faculty of Agriculture, Niigata University, 8050 Ikarashi 2-Nocho, Niigata, 950-2181 Japan; 2 Graduate School of Technology, Industrial, and Social Sciences, Tokushima University, 2-1 Minamijosanjima, Tokushima, 770-8506 Japan; 3 Graduate School of Science and Technology, Niigata University, Niigata, 951-8585 Japan

**Keywords:** Ecology, Animal migration, Behavioural ecology, Conservation biology, Ecological modelling

## Abstract

Radar is a powerful technology for surveys of avian movements. Validating the accuracy of radar detection is essential when establishing quantitative criteria for tracking bird trajectories and counting bird flocks. This study clarifies the positional and biological factors influencing the probability of detection (POD) and echo size on X-band marine radar. The bird trajectory for validation was obtained by ornithodolite at the same time as the radar scan. Distance was found to have a negative effect on POD and echo size, while elevation angle positively affected POD. Body mass and flock size positively affected POD and echo size. In predicting detection performance, the survey distance required to achieve 50% POD was 750 m or less for Grey-faced Buzzard, the lightest target species, but up to 1800 m for a pair of Bewick’s Swan. Our study provides survey and analysis procedures that allow for efficient validation using ornithodolites. Then, we identify the range settings that should be considered for target species and contribute to establishing criteria for quantitative radar bird surveys.

## Introduction

Spatial information on flying birds has long been of academic and social interest^1–3^. With the rapid increase in wind power generation projects since the 2000s^4^, collisions between birds and wind turbines have become a major worldwide concern^5–9^, increasing the need for detailed information on the density of birds in flight and greater accuracy for the assessment of the risk of bird collisions with infrastructure. Until the 2000s, visual observation was the predominant means of conducting spatial bird surveys for environmental impact assessments (EIA)^10^. With the subsequent development of technology offering more accurate methods, such as telemetry, ornithodolite, and radar, these have been introduced into EIA surveys^10^. Telemetry is excellent for obtaining species-specific flight parameters (e.g., height and speed); however, capture is necessary to attach telemetry equipment, and capture methods have not been established for all species. Furthermore, capture has restrictions on timing and location, and it may not be practicable to capture the birds being studied^10^. For example, most seabirds are only easily caught at their breeding site, which may be hundreds of km from where observations are to be made. In contrast, it has been practical to apply visual and radar surveys to a wider range of species over a wider range of seasons and locations. Few radar studies have obtained species-identified flight data^11^, yet radar allows monitoring at night and over long periods, which is difficult by visual observation^12,13^. In addition to these advantages, the ability to quantify the number of flying birds over a wide area^14,15^ has led to radar being widely used in risk assessment for wind power projects.

The detection of birds by radar in the 1940s led to widespread research and since the 1950s attempts have been made to monitor migratory birds and validate detection accuracy^16,17^. Currently, the primary radar systems used for spatial bird surveys in general are: weather, marine, and bird-specific radars (hereafter referred to as “bird radar”) such as MAX® and IRIS® offered by Robin Radar Systems^18,19^. Weather radar primarily uses C-band frequencies (also X and S) and scans three-dimensionally to estimate bird density and speed over a wide range (5–25 km)^18,20^. Marine radar uses X or S-band frequencies to scan two-dimensionally and track individual flights within a short, local range (0.1–3 km). Bird radar is an integrated system used primarily at airports, that includes X-band scanning and tracking software, and yields 3D trajectories at ranges up to 10 km^19^. In selecting the type of radar to be used for avian research, financial costs, data characteristics, and local radio laws regulating the use of such equipment must all be taken into consideration.

In incorporating radar into bird surveys, it is essential to identify factors that affect echo characteristics and quantification of flying birds to ensure survey accuracy^14,21,22^. Bird radar has echo-tracking software built into it, making it possible to align survey criteria between surveyors. However, there is a tradeoff between quality of the observations and radar availability due to high installation costs and strict radio regulations. In contrast, X-band marine radar, the least expensive and least regulated, has been used widely for bird surveys and validated for observation accuracy^12,14,22–24^. However, recent studies involving individual tracking of birds have focused on the trajectory output produced by the respective different tracking standards^14,15,21,22,25^. In general, the initial data obtained from the radar is two-dimensional signal intensity data (hereafter referred to as “raw data”). This raw data is converted as an echo image using radar manufacturer-specific standards and displayed on the terminal. The tracking software predicts and extracts flight trajectories from the echo data. Tracking algorithms vary from operator to operator and software to software, so previous validation studies are not necessarily universal. For example, if the number of non-detected echoes allowed in generating a trajectory is large, the trajectories appear over a wider area and are overestimated. Therefore, validation using raw data and echo images (pre-tracking data) is considered essential for a more accurate understanding of radar detectability, as well as important in developing and improving algorithms for more stable and accurate tracking software.

In recent studies generating trajectories of flying birds, echo images from marine radar have been used more generally than raw data^11,14,22,23,25,26^. The reason for this may be the difficulty in acquiring and handling raw data, as it cannot be acquired by a radar system alone but requires additional equipment, and the volume of raw data is much larger than that of images. Therefore, we have focussed on echo images, rather than raw data, in keeping with the reality of recent practice in radar surveying of birds, and because this simple approach can be implemented at minimal cost.

In this study, we clarify the effects of five factors (two location-related and three biological) on the probability of detection (POD) and the area on the display (recorded as number of pixels) occupied by a bird echo (a measure of the target’s size) obtained from X-band marine radar. The location factors were distance and elevation angle, and the biological factors were species differences in body size, flight mode (raptor employing soaring flight or non-raptor with flapping flight), and flock size (for waterfowl). We examined whether that the size of bird or flock being targeted and the ease of flapping behavior may affect the radar reflection intensity. By clarifying the factors that influence the echoes, we provide estimates of survey ranges and species-specific detection performance for quantitative bird surveys by means of marine radar.

## Results

### Characteristics of bird echoes extracted

We conducted radar surveys in Naruto City, Tokushima Prefecture, and Seiro Town, Niigata Prefecture in Japan (see Methods for site details). At the same time, we tracked individual birds with ornithodolite and obtained flight trajectories. Echoes at the exact timing and location of the ornithodolite-derived trajectories were selected as data for analysis.

At Naruto, 42 trajectories of 10 bird species were matched and 628 echoes and 212 non-detections were obtained from radar images. Distances ranged from 86.1 to 1192.2 m, the relative heights from the radar from -105.0 to 385.0 m, and elevation angles from -7.5 to 66.8 (Absolute angle from beam axis: 0.1 to 66.8). The wingspans and body mass of the 10 species were estimated from literature with values ranging from 0.91 to 1.60 m and 0.42 to 2.2 kg (Table 1). Six of the 10 species were raptors and were defined as soaring birds. This dataset was used to validate the species differences in radar echoes.

**Table 1:** The number of echoes and species-specific parameters used in the analysis.

Survey site	Scientific names	English names	Trajectories	Echoes	Non-detections	WS	BM	FM
Naruto	*Butastur indicus*	Grey-faced Buzzard	9	111	28	1.05^35^	0.42^35^	+
	*Accipiter gentilis*	Northern Goshawk	1	18	4	1.07^37^	0.93^37^	+
	*Buteo japonicus*	Japanese Buzzard	1	14	0	1.29^35^	0.86^35^	+
	*Pernis ptilorhyncus*	Oriental Honey Buzzard	2	32	3	1.26^36^	0.78^36^	+
	*Milvus migrans*	Black Kite	11	205	87	1.52^36^	0.82^36^	+
	*Pandion haliaetus*	Osprey	3	56	9	1.60^36^	1.58^36^	+
	*Corvus corone*	Carrion Crow	6	97	29	0.91^36^	0.57^36^	-
	*Larus crassirostris*	Black-tailed Gull	1	7	13	1.27^35^	0.56^35^	−
	*Phalacrocorax carbo*	Great Cormorant	5	58	9	1.40^36^	2.22^36^	−
	*Ardea alba*	Great Egret	3	30	30	1.44^36^	0.89^36^	−
	Total		42	628	212			
Seiro	*Cygnus (columbianus) bewickii*	Bewick’s Swan	14	680	665			

WS wingspan (m), BM body mass (kg), FM flight mode (a soaring bird or not).

At Seiro, 14 trajectories of Bewick’s Swan were matched and 680 echoes and 665 non-detections from radar images were obtained (Table 1). Distances ranged from 1180.0 to 4532.9 m, the relative heights from the radar from 109.5 to 487.0 m, and elevation angles from 2.1 to 14.9. Flock size varied from 2 to 39. This data set was used to validate the flock size difference in radar echoes. Bird species (e.g., crows and raptors) for which no clear formation of flight flocks could be identified were excluded from this analysis.

### Effect of species differences on echoes

We created generalized linear mixed models with distance from the radar, elevation angle, bird body mass, wingspan, and flight mode as explanatory variables, detection and non-detection (1–0), and echo size as response variables.

All models (ΔAIC < 2) differed significantly from the null model and are summarized in Table 2 (POD models: n = 840, echo size models: n = 628). Distance and body mass showed significant negative and positive effects in the best models with the lowest AIC for POD and echo size (Table 2). In contrast, elevation angle, wingspan, and flight mode had no or only small effects (Table 2).

**Table 2:** Effects of species characteristics on probability of detection and echo size.

	Variables					INT	ΔAIC	R^2^ m	R^2^ c	LRT
	D	A	BM	WS	FM					
POD (n = 840)	− 1.17***		0.76***			1.41	0.00	0.23	0.31	***
	− 1.16***		0.79***	− 0.06		1.41	1.87	0.23	0.31	***
	− 1.18***	− 0.03	0.76***			1.41	1.96	0.23	0.31	***
	− 1.17***		0.76***		− 0.02	1.41	1.99	0.23	0.31	***
Echo size (n = 628)	− 0.39***		0.57***			0.00	0.00	0.20	0.49	***

POD probability of detection, D distance, A absolute value of elevation angle, BM body mass, WS wingspan, FM flight mode, Int intercept, AIC Akaike’s Information Criterion, R^2^m marginal R^2^, R^2^c conditional R^2^, LRT likelihood ratio test, *p < 0.05 **p < 0.01 ***p < 0.001.

We estimated factors affecting POD and echo size by applying a generalized linear mixed model (GLMM) with tracks as a random factor.

**Fig. 1:**
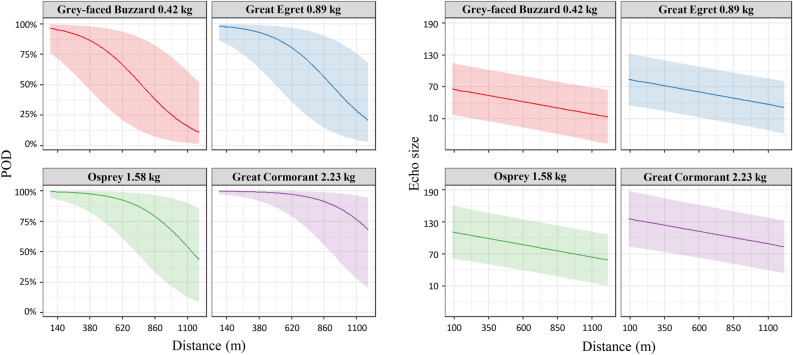
Prediction of probability of detection and echo area in relation to avian body mass. We predicted POD and echo size and their 95% prediction intervals per distance for four species of different body mass.

We predicted the POD and echo size with distance for four species with different body mass (Fig. 1). Distances with a 50% probability of detecting echoes were approximately 750 m for the smallest raptor in our study, Grey-faced Buzzard. For the largest bird, Great Cormorant*,* POD was approximately 70% at 1190 m, the maximum distance recorded in this survey. Echo size at 750 m, where POD exceeds 50% for Grey-faced Buzzard and Great Cormorant was predicted to be 26.2 and 139.3, respectively.

### Effect of flock size on echoes

We created generalized linear mixed models with distance from the radar, elevation angle, and bird flock size as explanatory variables, detection and non-detection (1–0), and echo size as response variables.

All models (ΔAIC < 2) differed significantly from the null model and are summarized in Table 3 (POD models: n = 1345, echo size models: n = 680). Distance has significant negative effect for POD and echo size. Elevation angle had significantly positive effects on POD, but no effect on echo size. Flock size significantly positively affected POD and echo size.

**Table 3:** Effects of flock size on probability of detection and echo size.

	Variables			Intr	ΔAIC	R^2^ m	R^2^ c	LRT
	D	A	F					
POD (n = 1345)	− 1.65***	0.99*		0.50	0.00	0.38	0.76	***
	− 1.81	0.72	0.84	0.53	0.01	0.44	0.75	***
	− 2.22***		1.08*	0.52	0.60	0.47	0.72	***
Echo size (n = 680)	− 0.40***		0.33*	0.15	0.00	0.19	0.32	***

POD probability of detection, D distance, A absolute value of elevation angle, F flock size, Int intercept, AIC Akaike’s Information Criterion, R^2^m marginal R^2^, R^2^c conditional R^2^, LRT likelihood ratio test, *p < 0.05 **p < 0.01 ***p < 0.001.

We estimated factors affecting POD and echo size by applying a generalized linear mixed model (GLMM) with tracks as a random factor.

**Fig. 2:**
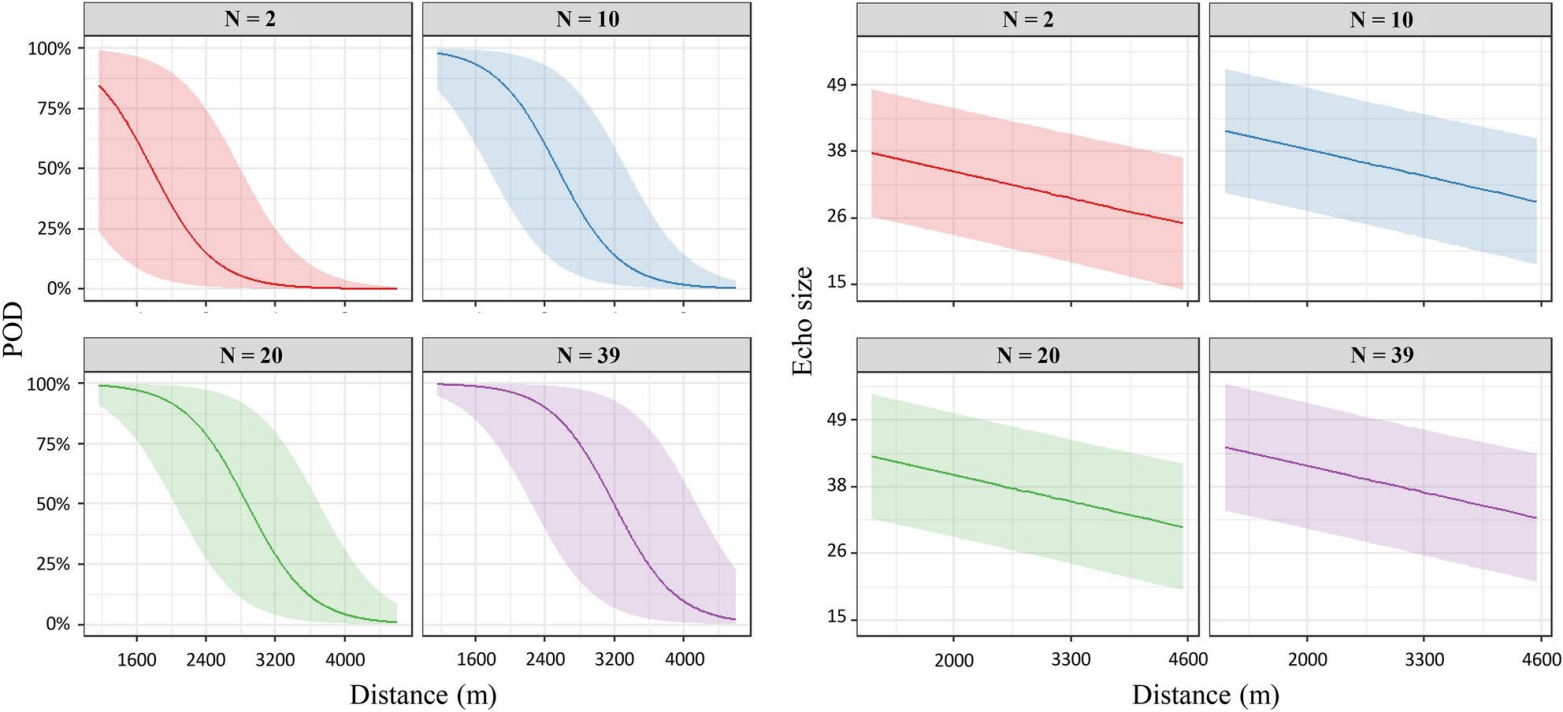
Prediction of probability of detection and echo size for various avian flock sizes. We predicted POD and echo size and their 95% prediction intervals per distance for different sizes of Bewick’s Swan flocks.

We predicted POD and echo size with distance for four Bewick’s Swan flock sizes (Fig. 2). Distances with a 50% probability of detecting echoes were approximately 1800 m for flocks of two (smallest flock size) and 3200 m for flocks of 39 (largest flock size). Echo size at 1800 m where the POD exceeds 50% in flocks of two and 39 was predicted to be 24.2 and 46.0.

## Discussion

### Validation of detection performance for bird surveys

Our echo image-based validation showed that distance and target size affected the detection performance of X-band marine radar with regards to birds in flight, which supports previous tracking-based validation^14,25,27^. For bird body size, body mass was a more promising predictor of echo detection and echo size than wingspan. If differences in biological and positional characteristics of target were not taken into account, radar survey results would underestimate echo detection and track length for lighter targets, smaller flocks, and more distant targets. Furthermore, echo detection was not underestimated for less flapping flight mode, such as birds of prey. Analysis using a data set that links real-time flapping behavior and the appearance of echoes will be necessary.

To achieve spatial quantifiability in radar bird surveys, proper survey ranges, tracking algorithms, and correction for the number of birds detected, are all essential processes^29^. Dokter et al. noted that many bird surveys overestimate radar detection performance and fail to set appropriate radar ranges^14^. Our suggestion, based on this study, is that investigators should set a radar observation range with a predicted POD of at least 50% for tracking algorithms that allow up to 50% non-detection of echoes. When comparing flight counts across species, correcting for the number of birds detected needs to be done carefully. This is because species and distance differences affect detection performance independently. However, species identification of tracks produced by radar is not commonly done^13^, making it difficult to make accurate corrections based on species characteristics. Therefore, in actual surveys, it is necessary to list bird species known to occur in the survey area based on visual observation and to indicate which species may be either under or over-estimated. Maximum observing ranges should be set for targets that are hard to detect, such as lighter species or species that do not form flocks; for example, in research areas where birds of raptor and waterfowl types coexist, a range for the former would be appropriate.

A more constructive discussion of height can be made if it is considered an elevation angle, since the range of observable heights varies with elevation angle and distance. Previous studies (along with this study) have shown a positive effect on POD at elevation angles below 15°^16,27^. An increase in elevation angle might be assumed to increase the radar cross-section of a bird in flight with its wings spread horizontally. However, an increase in elevation angle greater than 15° could have offset detection performance due to beam shape effects.

A further factor requiring validation is the difference in power output of the radar. For the X-band marine radar, 25 kW is the most frequently used, which theoretically has a higher detection capability than the 12.5 kW radar used in this study^14,22–26^. According to the radar equation^30^, doubling the transmitted power will increase the detection range by a factor of –1.2 and thus potentially increase the area surveyed by − 40%.

### Methodological developments in radar bird surveys

To enhance radar validation studies, bird echo matching issues need to be addressed. In our study, the use of ornithodolites made it easy for us to proceed with bird matching because continuous flight trajectories were obtained in addition to the precise time and location of the target. Because the ornithodolite tracking distance is 1–3 km for many bird species^31,32^, researchers can deliberately set the survey area of the radar scan area to overlap.

We can collect validation data for more species, under more environmental conditions, which will make the validation more robust. We therefore recommend the use of ornithodolites for the collection of observational data. Observations for matching have previously been acquired by line transect and telemetry^14,25^, neither of which is a highly efficient data collection method. Line transects, are limited by the direction that can be surveyed, in addition to the need to resolve the problem of inaccuracy in direct observation^14^. In the case of telemetry, it is impossible for surveyors to capture every species that appears in the survey area. Furthermore, whether or not tagged individuals will fly over the radar observation area cannot be predicted. In addition, if the ornithodolite positioning interval is matched to the radar rotation speed, it is also possible to collect flight-related features (speed and angle), which can contribute to creating future species classifiers. However, we note that ornithodolites make nighttime observation impossible for models not equipped with night vision scopes, that tracking distance is attenuated by rain and fog, and that species identification in parallel with observation tracking is essential.

Detection performance varies with position (distance and/or elevation angle) and body mass and flock size for different bird species. Species identification is essential for accuracy in quantifying the detection frequency of echoes. The development of translating radar-derived information into bird attributes (species, flock size, and behavior) is a challenging subject. However, Rosa et al. (2016) used machine learning and factors including air speed, angle, and echo shape to classify the trajectories they observed into five classes (other birds versus clutter, herons, gulls, swallows, and storks)^11^. The accuracy, sensitivity, and specificity rate were 0.75 or higher for all five classifications. Equations relating outputs such as POD and echo size to distance and object size are also useful information for species and flock size estimation. However, our analysis only covers the occurrence and magnitude of a single echo, even in a flock, so additional analysis is needed to apply it to a flock of complex shapes, such as several hundred waterfowl. Clarifying the echo parameters and POD among various species and finding consistency among radar models are important for developing a more robust species classifier.

Survey standards, based on universal performance test results, are needed to enable radar surveying to be adopted more widely. To this end, the definition of detection potential based on image data before echo tracking and the collection of validation data using ornithodolite would be effective. We expect validation data to be advanced with more bird species, environments, and radar models. Radar surveys based on more robust criteria can contribute to environmental impact assessments prior to construction of wind farms and can help to reduce as much as possible conflicts between wildlife and humans using the same air space as a shared resource.

## Methods

### Radar system

We used an X-band marine radar (MDC-7910, manufactured by Koden Electronics, Tokyo, Japan; magnetron-amplified radiation, 12.5 kW power output, 6.5 feet horizontally scanning T-bar antenna, 1280 × 1024 monitor) to observe flying birds. The half-power point of beam width was 1.2° horizontally and 22° vertically (11° up and down).

**Fig. 3:**
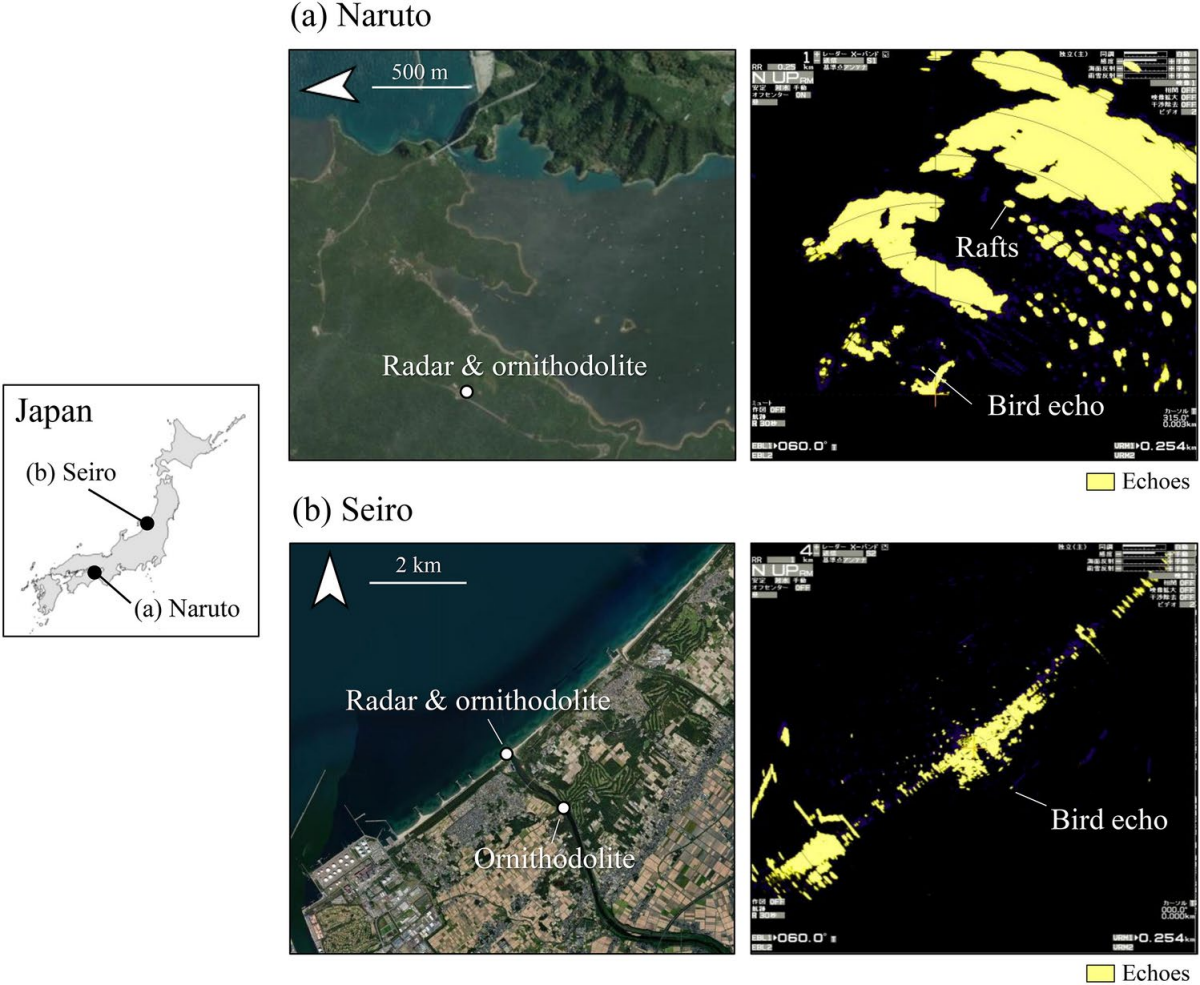
Radar locations and X-band radar images. The radars are located at (**a**) Naruto (N34.23273, E134.60334) and (**b**) Seiro (N38.02204, E139.27741). The range settings are 1 km and 4 km in Naruto and Seiro. The map was created in Esri ArcGIS 3.0 software (https://www.esrij.com/products/arcgis-pro/). *Source *of satellite images: Esri, Maxar, Earthstar Geographics, and the GIS User Community.

Radar observations were made at two locations (Fig. 3). Naruto, in Tokushima Prefecture (N34.23273, E134.60334), is a well-known spot for observing migratory birds of prey, such as Grey-faced Buzzard and Oriental Honey Buzzard. At Naruto the radar is located on a cliff 110 m above sea level. The range where the beam is unobstructed by terrain is the sea to the east and south (Fig. 3a). As this is an inland sea and the wind conditions on the survey day were calm, no wave clutter appeared. Seiro, in Niigata Prefecture (N38.02204, E139.27741), is a well-known spot for observing migratory waterfowl. At Seiro the radar was installed on a sandy beach 8 m above sea level (Fig. 3b). We removed the terrain clutter on the inland side by placing the radar in the shade of coastal forest. This procedure allowed for stable display of the echoes of birds in flight, even over land. The range for the radar system was set to 1 km at Naruto and 4 km at Seiro. Radar scans were performed at a rotation speed of 48 rpm, gain sensitivity of 75, and ground reflection rejection of 0. Echo images, colored yellow by default, were recorded every second. Radar surveys were conducted at Naruto from September 28th to October 2nd, 2022, and at Seiro on October 25th and 26th, 2022.

### Bird tracking by Ornithodolite

We conducted ornithodolite tracking simultaneously with radar scans so as to match echoes to birds. By using an ornithodolite (Vector 21 Aero, manufactured by Safran Vectronix, Heerbrugg, Switzerland, 1σ distance error ± 5 m, 1σ elevation error ± 0.2°, 1σ azimuth error ± 0.6°), the azimuth, elevation, and oblique distance of each flying object could be measured, and its 3-D position calculated. Ornithodolite observations were made at the same locations as the radar observations. In addition, at Seiro, an observation site was added approximately 1.5 km inland from the radar site (Fig. 3). We tracked birds at 2.3-s intervals using an ornithodolite support system (LMS 1.0, manufactured by Kyokuto Boeki Kaisha, Tokyo, Japan), and recorded species and flock size. When tracking a flock, we targeted bird in the center of the flock and stopped tracking when the flock had apparently dispersed or merged with another flock.

Using Ornithodolite, we can track raptors and seabirds up to approximately 1.5 km and waterfowl up to approximately 2.5 km. Variation in the POD of birds by radar within the observation range of the ornithodolite is ideal for validation. At Naruto, the decrease in bird detection rate with distance from radar within the observation range of the ornithodolite set at the same location as the radar was preliminarily confirmed. At Seiro, where swans were targeted, we set our ornithodolite survey sites at different locations from the radar. The reason is that the swans have a long detectable range and setting them at the same location as the radar would not provide the sufficient distance for validation. Tracking data is available in Supplementary Information 1, 2.

### Extraction of bird echoes and image processing

**Fig. 4:**
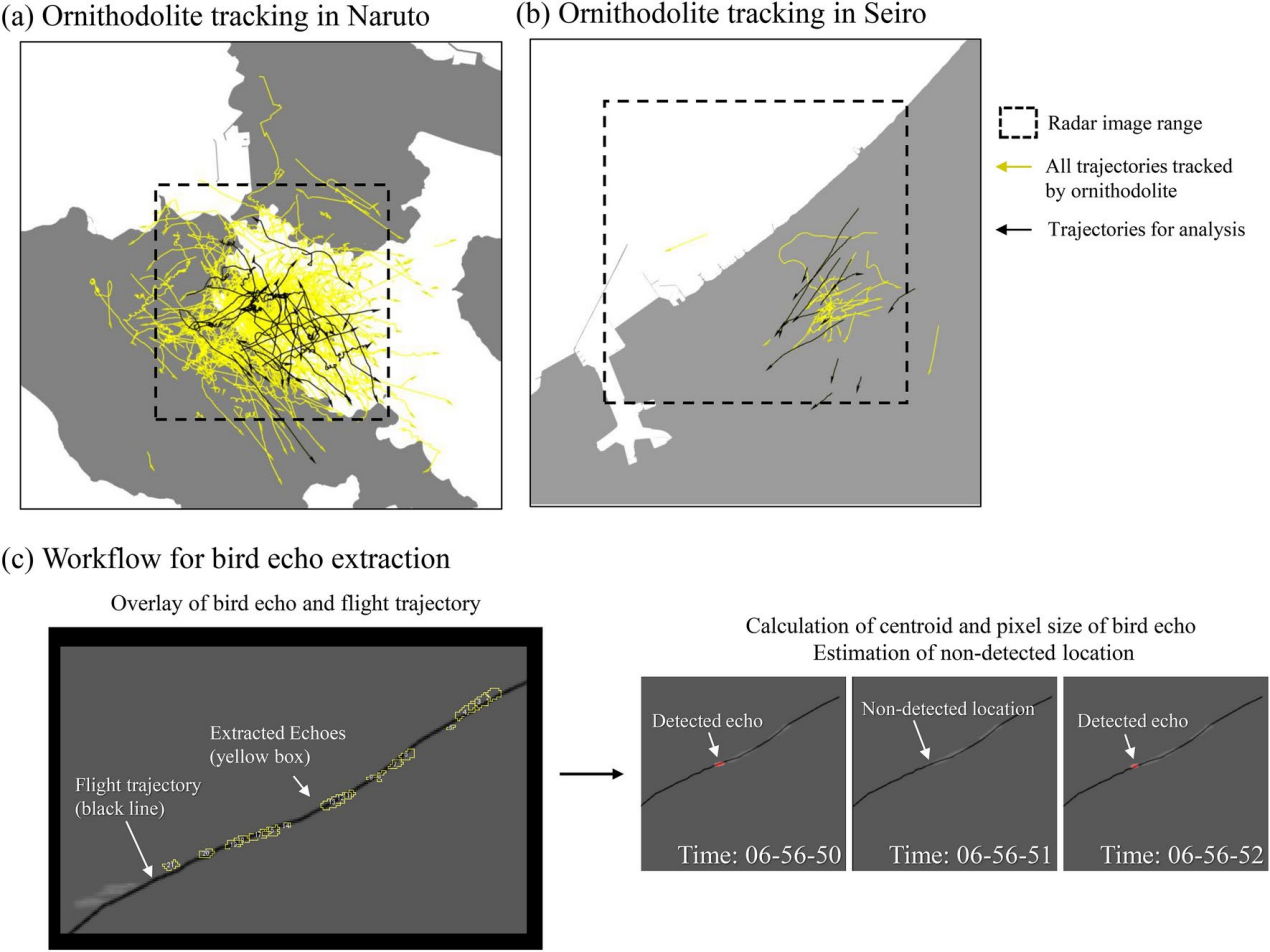
Echo extraction procedure for analysis. We tracked the flight trajectory using ornithodolite in (**a**) Naruto and (**b**) Seiro. (**c**) We selected echoes for analysis by extracting bird echoes from a radar image sequence and manually identifying and drawing the trajectories. The black line indicates the flight trajectory, and the yellow box indicates the echoes. The coordinates of the center gravity and echo size of the bird echoes were calculated. The coordinates of the bird in the non-detected image were estimated from the bird echoes before and after. The map was created in Esri ArcGIS 3.0 software (https://www.esrij.com/products/arcgis-pro/). Land polygon source: National Land Numerical Information download service (Administrative Zones Data). Ministry of Land, Infrastructure, Transport and Tourism (http://nlftp.mlit.go.jp/ksj-e/gml/datalist/KsjTmplt-N03.htm).

The extraction procedure of bird echoes and the procedure for image processing are complex (see Fig. 4).

We used ESRI ArcGIS pro software (version 3.0) to select bird flight trajectories for matching bird echoes. We identified tracks passing over terrain clutter by overlapping the ornithodolite-derived tracking maps with radar images. We omitted images including these trajectories from the analysis target because bird echoes on terrain clutter are masked and cannot be extracted.

We examined visually the radar images frame-by-frame to check for the movement of echoes, and identified trajectories where the time, place, and flight direction from ornithodolite tracks matched the movements of the echoes. We then targeted the selected trajectories and echoes for analysis. In the echo samples from a single trajectory, the analysis excluded echoes that crossed or merged with other echoes or separated into multiple echoes. Thus, the flocks analyzed are cohesive as a single echo.

We used Image J (US National Institutes of Health, Bethesda, Maryland, USA) for processing radar images. The radar image was monochromatically transformed, and we extracted echoes with luminance greater than 100 (range 0–255) and an area greater than five pixels. We calculated the center-of-gravity coordinates and echo sizes of extracted echoes. For the location of the bird in the image where no echo (non-detection coordinates), we could not directly calculate the centre of gravity because there were no echoes. Therefore, we estimated the non-detection coordinates to be equally spaced according to the number of radar scans between the extracted echoes. Estimation of non-detected coordinates was kept within the range of detected coordinates because outside the detection echo coordinates, unpredictable factors such as small terrain shadows or sudden changes in flight altitude may be involved in non-detection.

Observation distances were calculated from the centroid and radar coordinates of echoes. Elevation angles were calculated from the distance described above and the median altitude of the trajectory obtained by ornithodolite tracking. The absolute value of the elevation angle was used because radiated and received power is reduced at angles above and below the horizontal beam axis.

### Modeling

We constructed generalized linear mixed models (GLMM) using the “lme4” and “lmerTest” package for R ver4.2.1 to explain the echo’s POD and echo size^33–35^. The POD was modeled using a binomial error structure, and the echo size was modeled using normal errors. The random effect structure was the trajectory ID in both models.

As explanatory variables to validate species differences in POD and echo size, we used wingspan, body mass, and flight type (binary indication whether the species was a soaring bird or not) (Table 1). For wingspan and body mass, values found in the literature were used as references^36–38^. Flight mode was used as a factor expected to reduce reflection intensity and POD for species with flight modes with less flapping^28^. The number of birds in each flock was used to validate the effect of flock size on POD and echo size. We used distance and elevation angle for both models as the factors related to the echo position. The datasets used in the analysis are available in Supplementary Information 1, 3, 4.

Echo size and all explanatory variables were standardized to a mean of 0 and a variance of 1. All combinations of explanatory variables (not considering interactions) were tested using the “MuMIn” package^39^, and the AIC was calculated. Models with ΔAIC < 2 were deemed worthy of consideration, and marginal R^2^ and conditional R^2^ were calculated after likelihood ratio tests with intercept-only model. POD and echo size were predicted using the model with the highest Marginal R^2^ in the models with ΔAIC < 2.

## Supplementary Information

Supplementary Information 1.

Supplementary Information 2.

Supplementary Information 3.

Supplementary Information 4.

## Data Availability

The datasets generated and analysed during the current study are available in the Supplementary files 1, 2, 3 and 4. The radar images are available from the corresponding author on request.
